# Wan-Nian-Qing, a Herbal Composite Prescription, Suppresses the Progression of Liver Cancer in Mice by Regulating Immune Response

**DOI:** 10.3389/fonc.2021.696282

**Published:** 2021-07-08

**Authors:** Xinrui Zhang, Xin Liu, Yue Zhang, Anhui Yang, Yongfeng Zhang, Zhijun Tong, Yingwu Wang, Ye Qiu

**Affiliations:** ^1^ Department of Pharmacy, Changchun University of Chinese Medicine, Changchun, China; ^2^ School of Life Sciences, Jilin University, Changchun, China; ^3^ R&D Department, Jilin Tianlitai Pharmaceutical Co. Ltd, Baishan, China

**Keywords:** Wan-Nian-Qing prescription, liver cancer, apoptosis, immune response, Nrf2

## Abstract

The Wan-Nian-Qing prescription (WNQP), an herbal composite containing *Ornithogalum caudatum*, has been used in China for several years for cancer treatment. However, the mechanism of its pharmacological action against liver cancer is not clear. This study aimed to investigate the role of WNQP in inhibiting tumor growth in hepatocellular carcinoma model mice and determine its mechanism of action. We established HepG2- and SMMC-7721-xenografted tumor models in nude mice and BALB/c mice. The mice were administered WNQP for 2 weeks. The bodyweight of each mouse was monitored every day, and the tumor size was measured using vernier caliper before each round of WNQP administration. After the last dose, mice were sacrificed. The tumors were removed, lysed, and subjected to proteome profiling, enzyme-linked immunosorbent assay, and western blotting. The liver, spleen, and kidney were collected for histopathological examination. The effects of WNQP against liver cancer were first systematically confirmed in HepG2- and SMMC-7721-xenografted nude mice and BALB/c mice models. WNQP inhibited tumor growth, but failed to affect bodyweight and organ structures (liver and spleen), confirming that it was safe to use in mice. In BALB/c mice, WNQP regulated immune function, inferred from the modulation of immune-related cytokines such as interleukins, interferon, tumor necrosis factors, and chemokines. Further results confirmed that this regulation occurred *via* the regulatory effects of WNQP on Nrf2 signaling. WNQP can inhibit the growth of HepG2- and SMMC-7721-xenografted tumors in nude mice and BALB/c mice. This effect manifests at least partially through immunomodulation mediated apoptosis.

## Introduction

Liver cancer is the sixth most common type of malignant tumor in the world and the second most common cause of tumor-related deaths (1). Epidemiological data from the United States in 2018 shows that between 2011 and 2015, the mortality of patients suffering from liver cancer increased by 2.7% per year among women and 1.6% per year among men (2). In China, the situation is even worse. Although treatment strategies have improved significantly, they mainly involve surgery, chemotherapy, radiotherapy, and immunotherapy (3). Unfortunately, most patients are not eligible for these curative treatments when they are at the advanced stages of the disease. To facilitate a breakthrough in the safety and efficacy of liver cancer treatment, there is an urgent need to develop new treatments to help increase patient life expectancy and the clinical benefits.

Oxidative stress is defined as the excessive production of reactive oxygen species (ROS) (4) and perturbation of the cell’s redox balance (5) in a manner that cannot be counteracted by antioxidants. Oxidative stress factors can damage DNA and DNA repair enzymes, activate proto-oncogenes, disrupt cell signaling molecules, and ultimately lead to cancer. Nuclear factor-erythroid 2-related factor 2 (Nrf2) is a basic leucine zipper (bZIP) protein that regulates the expression of antioxidant proteins that protect against environmental oxidative stress (6, 7). In the nucleus, Nrf2 binds to the antioxidant response element (ARE), resulting in the transcription of antioxidant genes such as heme oxygenase-1 (HO-1) and superoxide dismutase (SOD) (8). The improvement of antioxidant capacity is contributed to improve the immune response of the host (9).

In China, traditional Chinese medicine has been used in clinics for a long time because of its wide efficacy and low side effects. The Wan-Nian-Qing-Prescription (WNQP) is a kind of compound traditional Chinese medicine commonly used in the treatment of malignant tumor, mainly composed of *Ornithogalum caudatum Jacq* (OC) (30%), *Scutellaria barbata D.Don* (SB) (15%), *Reynoutria japonica Houtt* (RJ) (6.70%), *Curcuma aromatica Salisb* (CAS) (7.50%), *Hedyotis diffusa Willd* (HD) (15%), *Panax ginseng C.A.Mey* (PG) (7.50%), *Salvia miltiorrhiza Bunge* (SMB) (7.50%), *Astragalus propinquus Schischkin* (APS) (15%), *Buthus martensii Karsch* (BMK) (3%), and *Scolopendra subspinipes* (SS) (3%) (**Table 1**). WNQP has been used in the combination of chemotherapy for liver cancer, lung cancer and gastric cancer for several years (10). In the theory of traditional Chinese medicine, OC and RJ both have the functions of dieresis and removing moisture of the body; SB has the function of clearing away heat; HD has the functions of detoxification and anti-cancer; CAS can enhance the function of the stomach; PG, SMB and APS can enhance human immunity; while BMK and SS have the function of killing cancer cells. Moreover, in the research results of modern pharmacy, OC is mainly composed of saponins, polysaccharides, terpenes and flavonoids and has a variety of pharmacological activities, such as anti-tumor, anti-inflammatory, and immunity-enhancing effects, as have been previously reported (11, 12). The total saponins present in the OC can inhibit the growth of HepG-2 cells by regulating mitochondrial function (13). HD can inhibit tumor growth *in vivo* and *in vitro* (14). APS combined with cisplatin can inhibit the growth of Lewis lung cancer cells and reduce the expression levels of CD44, CD62P and OPN protein in tumor tissue (15). WNQP has been shown to reduce the adverse reactions caused by mFOLFOX6 chemotherapy in patients with late-stage gall bladder cancer by improving their immune function (16, 17). However, the effects of WNQP against liver cancer and the mechanisms underlying the same have not been systematically reported, especially in animal models.

**Table 1:** The English name, full botanical plant name and the ratio of the ingredients of Compound WNQP Capsule.

Full botanical plant name	English name	Ratio
*Ornithogalum caudatum* Aiton	*Ornithogalum caudatum*	33.40%
*Scutellaria barbata* D.Don	Half Lotus/Barbate Skullcap	15%
*Reynoutria japonica* Houtt.	*Polygonum cuspidatum*	6.70%
*Curcuma aromatica* Salisb.	*Radix curcumae*	7.50%
*Hedyotis diffusa* Willd.	Oldenlandia	15%
*Panax ginseng* C.A.Mey.	Ginseng	7.50%
*Salvia miltiorrhiza* Bunge	Salvia mill	7.50%
*Astragalus propinquus* Schischkin	*Astragalus membranaceus*	15%
*Buthus martensii* Karsch*	Scorpion	3%
Scolopendra subspinipes*	Centipede	3%

*Not plants.

In this study, we systematically investigated the effects of WNQP against liver cancer on HepG2- and SMMC-7721-xenografted mouse models. We assessed the roles of WNQP in regulating the immune response *via* modulation of the oxidative stress response. We specifically focused on the regulation of Nrf2 signaling as a possible mechanism underlying the effects of WNQP against liver cancer. Based on our experiments, we provide novel evidence in support of the therapeutic formula WNQP in treating liver cancer patients.

## Materials and Methods

### WNQP Information

The compound WNQP capsule, with national medicine approval number B20020717 and implemented in accordance with the China National Food and Drug Administration National Drug Standard (WS-5696 (B-0696)-2014Z), is a preparation comprising Chinese traditional medicines. We obtained the compound WNQP capsule with drug batch number 160301 from the Jilin Tianlitai Pharmaceutical Co., Ltd. For the *in vitro* experiment, WNQP was extracted with double distilled (DD) water at the ratio of 1:10 at 60°C for 4 h and stored at 4°C.

### Cell Culture

The liver cancer cells, HepG2 (American Type Culture Collection, USA) and SMMC-7721 (China Center for Type Culture Collection, China), were cultured in Dulbecco’s Modified Eagle Media (DMEM; Gibco, Grand Island, NY) containing 10% fetal bovine serum (FBS; Zhejiang Tian hang bio Polytron Technologies Inc.), and 1% penicillin and streptomycin at 37°C under 95%/5% air/CO_2_ conditions.

### Animal Models

The mice used for experiments were housed in barrier facilities (temperature: 23 ± 1 °C and humidity: 50 ± 10%) under a 12 h/12 h light/dark cycle. They had free access to food and water. The animal experiments were carried out under the supervision of the Institutional Animal Care and Use Committee of Jilin University (NO. 2017SY0601).

#### HepG2- and SMMC-7721-Xenografted Tumor Models in BALB/c Nude Mice

Five-week-old male BALB/c nude mice (SCXK(JI)-2016-0003; purchased from Wei-tongli-hua Laboratory Animal Technology Company, Beijing, China) were injected subcutaneously with 3–4 × 10^6^ cells of either the HepG2 or SMMC-7721 lineage. When the tumor volume reached 100 mm^3^, the tumor-bearing mice were divided into three groups (*n* = 6/group) for both the HepG2- and SMMC-7721-xenografted tumor models; the first group was given physiological saline (*n* = 6), the second group was given 0.3 g/kg of WNQP (*n* = 6), and the third group was given 0.6 g/kg of WNQP (*n* = 6). All doses of saline/WNQP were administered orally, every day for 2 weeks. The bodyweight of each mouse was monitored every day, and the tumor size was measured using vernier calipers before each round of saline/WNQP administration. The tumor volume was calculated using the following formula:

$$\text{V}(\text{m}{\text{m}}^{3})=0.5\times (\text{larger}\;\text{diameter}\times \text{smaller}\;\text{diamete}{\text{r}}^{2})$$

After the final dose, the mice were sacrificed. The tumors were then removed, lysed, and subjected to proteome profiling and western blotting. The liver, spleen, and kidney were collected and fixed in 4% paraformaldehyde (PFA) for histopathological examination.

#### HepG2- and SMMC-7721-Xenografted Tumor Models in BALB/c Mice

Male BALB/c mice (18–22 g, specific pathogen-free [SPF] grade) were purchased from the Yis Laboratory Animal Technology Co., Ltd., Changchun, China (SCXK(JING)2016-0011). After adaptive feeding, they were injected intraperitoneally with cyclophosphamide (CTX; 50 mg/kg) for three consecutive days. Fresh tumor samples obtained from the HepG2 and SMMC-7721 heterotopic tumor models in nude mice were cleaned and sliced with a scalpel to obtain 2 mm × 2 mm × 2 mm sections. The tumor masses were implanted in the right dorsum (near the hind leg) of the BALB/c mice, and tumor growth was monitored daily. When the tumors attained a certain volume, the mice were randomly divided into three groups (*n* = 6/group); the first group was given physiological saline (*n* = 6), the second group was given 0.3 g/kg of WNQP (*n* = 6), and the third group was given 0.6 g/kg of WNQP (*n* = 6). All doses of saline/WNQP were administered orally, every day for 4 weeks. To avoid the restoration of immune function in mice, CTX (50 mg/kg) was injected once a week. The day after the last administration, blood was collected from the hearts of the mice, after which the mice were anesthetized using sodium pentobarbital and photographed. After euthanasia of mice, the tumors were removed, lysed, and subjected to proteome profiling and western blotting. The liver, spleen, and kidney were collected and fixed in 4% PFA for histopathological examination.

### Nrf2-siRNA Transfection of Spleen Cells

The primary culture of spleen cells was seeded onto 6-well plates, in accordance with previously described protocol (18). The cells were transfected with 20 nM Nrf2-siRNA (5′-GGATGAAGAGACCGGAGAA-3′; R100438, RiboBio, China) for 30 min using the riboFECT™CP Reagent (RiboBio), in accordance with the manufacturer’s protocol. Following transfection, the cells were treated with 0.5 mg/mL of WNQP for 4 h and then harvested. The expression of Nrf2, HO-1, HO-2, SOD-1, and glyceraldehyde-3-phosphate dehydrogenase (GAPDH) was analyzed using western blots.

### Histopathological Examination

Liver, spleen, and kidney tissues were washed with water and dehydrated using different concentrations of alcohol. We used xylene to clear the tissues and then embedded the samples in paraffin wax. After the paraffin solidified, sections were cut using a microtome (Leica, Wetzlar, Germany) and were stained with hematoxylin and eosin (H&E) (19). Images were captured using an Eclipse TE 2000-S fluorescence microscope (Nikon Corp., Tokyo, Japan).

### Antibody Chip Analysis

The apoptotic factors expressed by the tumors were collected from BALB/c nude mice and detected using cytokine chip analysis. Briefly, total protein was extracted from tumor tissue and its amount was determined using a BCA Protein Assay Kit (Merck Millipore, Billerica, MA, USA). An Apoptosis Array Kit (ARY009, R&D Systems, Millipore, USA) was used to assess the presence of 35 factors in the total protein, following protocols outlined by the manufacturer.

The tumors obtained from the BALB/c mice were used to detect 308 mouse proteins. Total protein was extracted from tumor tissue using ice-cold tissue protein extraction reagent containing 0.5% protease inhibitor cocktail, phenylmethylsulfonyl fluoride, and phosphatase inhibitor cocktail. The total protein amount was determined using a BCA Protein Assay Kit. A RayBio^®^ L-Series Mouse Antibody Array 308 Glass Slide Kit (RayBiotech, AAM-BLG-1, USA) was used to assess the presence of 308 factors in the total protein, in accordance with the manufacturer’s protocols.

### Immune Cytokine Detection

Blood from BALB/c mice was collected from the caudal vein before performing euthanasia. The serum levels of interleukin (IL)-2 (42903), IL-10 (CK-E20005), IL-31 (44865), interferon-γ (IFN-γ) (42918), tumor necrosis factor (TNF)-α (CK-E20220), TNF-β (42867), and chemokine (C-C motif) ligand 28 (CCL28) (44866) were detected using specific enzyme-linked immunosorbent assay (ELISA) kits, in accordance with the manufacture’s protocol (The Source Leaf Biological Technology Co. Ltd., Shanghai, China).

### Western Blot Analysis

The tumors from BALB/c nude mice and spleens from BALB/c mice were homogenized along with a tissue protein extraction reagent. The total protein content was determined using a BCA Protein Assay Kit (Merck Millipore, Billerica, MA, USA). We loaded 40 μg of protein per sample and separated them using sodium dodecyl sulfate-polyacrylamide gel electrophoresis. The separated proteins were then transferred onto polyvinylidene fluoride membranes (0.45 μm, Merck Millipore, Billerica, MA, USA). Subsequently, the membranes were blocked and incubated with the appropriate primary antibodies: bcl2-associated X (Bax) (21 kDa) (ab32503), B-cell lymphoma-extra-large (Bcl-xL) (26 kDa) (ab32370; Abcam, Cambridge, MA, USA), Nrf2 (97 kDa) (12721S; Cell Signaling Technologies, Danvers, MA, USA), heat shock protein 27 (HSP27) (27 kDa) (bs-0730R), heat shock protein 60 (HSP60) (58 kDa) (bs-0191R), heat shock protein 70 (HSP70) (70 kDa) (bs-0126R), HO-1 (32 kDa) (bs-2075R), HO-2 (36 kDa) (bs-1238R), SOD-1 (17 kDa) (bs-10216R), GAPDH (37 kDa) (bs-0755R; Bioss Antibodies, China), overnight at 4°C. The membranes were then incubated with the appropriate secondary antibody, either a horseradish peroxidase (HRP)-conjugated goat anti-rabbit immunoglobulin G antibody (SH-0032; Beijing Dingguo Changsheng Biotechnology Co., Ltd., Beijing, China) or an HRP-conjugated goat anti-mouse immunoglobulin G antibody (sc-2005; Santa Cruz Biotechnology, Inc., Texas, USA), for 4 h at a dilution of 1:2,000. Finally, the blots were visualized using electrochemiluminescence detection kits (Merck Millipore, Billerica, MA, USA) and quantified using ImageJ software (NIH, Bethesda, MD, USA).

### Statistical Analysis

All data are presented as the mean ± standard deviation (SD). One-way analysis of variance and post-hoc multiple comparisons (Dunn’s test) were performed where applicable using the Statistical Package for Social Sciences (SPSS) v16.0 software (IBM Corporation, Armonk, NY). The statistical significance threshold was set at *P < *0.05.

## Results

### WNQP Inhibits HepG2- and SMMC-7721-Xenografted Tumor Growth in Nude Mice

**Figure 1:**
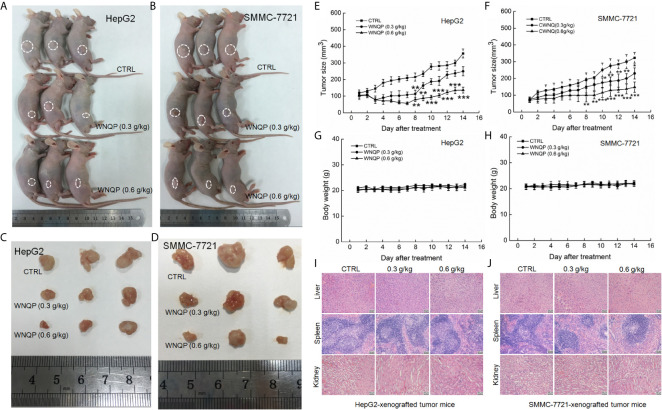
WNQP inhibited HepG2- and SMMC-7721-xenograft tumor growth in BALB/c nude mice. BALB/c athymic nude mice inoculated with HepG2 and SMMC-7721 cells were treated with WNQP (0.3 and 0.6 g/kg dissolved in 0.9% saline solution) or vehicle solvent (0.9% saline solution) for 14 days. **(A, B)** Tumor-bearing nude mice and **(C, D)** tumor tissues collected from control (CTRL) and WNQP-treated groups (*n* = 3). **(E, F)** Tumor volumes were measured every day. Tumor sizes are expressed as the mean ± SD (*n* = 6). *P < 0.05, **P < 0.01 and ***P < 0.001 *versus* the control group. **(G, H)** Mean ( ± SD) bodyweights of mice in the WNQP-treated and CTRL groups (*n* = 6). **(I, J)** H&E staining of liver, spleen, and kidney tissues from nude mice (*n* = 3) (20× magnification, scale bar: 50 μm).

The nude mice with tumor xenografts were used to investigate the anti-tumor activity of WNQP. A 14-day treatment regimen of WNQP administered at either 0.3 or 0.6 g/kg significantly inhibited the growth of tumors, but more so for the latter dose (HepG2-xeongrafted tumors: *P <*0.001, 136.5 mm^3^ *vs.* 357.0 mm^3^, **Figures 1A, C, E**; SMMC-7721-xenografted tumors: *P <*0.001, 148.2 mm^3^ *vs.* 323.3 mm^3^, **Figures 1B, D, F**). WNQP showed no significant effects on the bodyweights (**Figures 1G, H**) and organ structures of the liver, spleen, and kidney (**Figures 1I, J**) in either HepG2- or SMMC-7721-xenografted nude mice.

**Figure 2:**
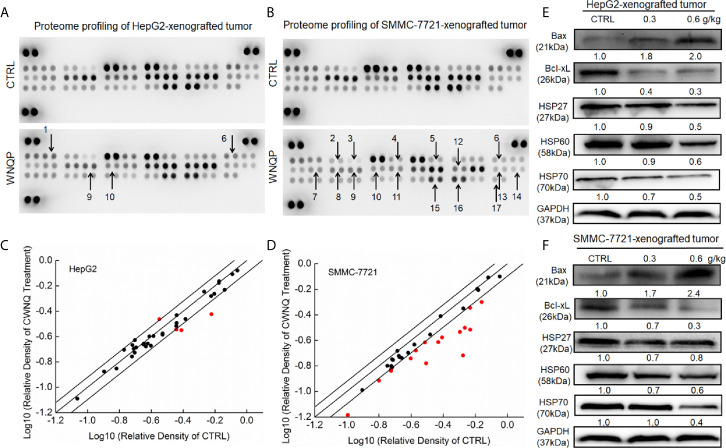
The effects of WNQP on 35 types of cytokines in nude mice tumors, detected using an Apoptosis Array Kit (*n* = 3). The arrows indicate factors marked for further detection: 1. Bax, 2. Bcl-2, 3. Bcl-xL, 4. cleaved caspase-3, 5. cIAP, 6. clusterin, 7. TRAIL R2/DR5, 8. FADD, 9. Fas, 10. HIF-1α, 11. HO-1, 12. HSP 60, 13. HtrA2, 14. livin, 15. SMAC, 16. survivn, 17. XIAP. **(A, B)** Scatter diagram of 35 cytokines. The relative density is the ratio of the absolute value and reference spot value. The red dots indicate factors with a change of >20% relative to control mice. **(C, D)** WNQP strongly enhanced the expression levels of Bax and suppressed the expression levels of Bcl-xL, HSP27, HSP60, and HSP70 in tumor tissues. Quantitative protein expression data were normalized to the corresponding GAPDH levels, and the average fold changes in band intensity are marked **(E, F)** (*n* = 3).

To systematically investigate the possible mechanisms underlying the inhibitory effects of WNQP on HepG2- and SMMC-7721-xenografted tumor growth in nude mice, cell cytokines related to apoptosis and oxidation were detected using a Proteome Profiler Apoptosis Array Kit. When HepG2- and SMMC-7721-xenografted tumors from nude mice were assessed, WNQP at 0.6 g/kg was found to strongly regulate the levels of cytokines related to apoptosis and oxidation, including Bax, Bcl-2, Bcl-xL, cleaved caspase-3, cellular inhibitors of apoptosis (cIAP), clusterin, TNF-related apoptosis-inducing ligand (TRAIL) R2/death receptor (DR) 5, Fas-associated protein with death domain (FADD), Fas, hypoxia-inducible factor (HIF)-1α, HO-1, HSP60, high temperature requirement protein 2 (HtrA 2), livin, supramolecular activation complex (SMAC), survivn, and X-chromosome-linked inhibitor of apoptosis (XIAP) (**Figures 2A–D** and **Table S1**).

Western blot analysis revealed that WNQP increased the expression levels of Bax, and reduced the expression levels of Bcl-xL, HSP27, HSP60, and HSP70 in HepG2- (**Figure 2E**) and SMMC-7721-xenografted tumors in nude mice (**Figure 2F**).

### WNQP Inhibits HepG-2 and SMMC-7721-Xenografted Tumor Growth in BALB/c Mice *via* Regulation of Immune Function

**Figure 3:**
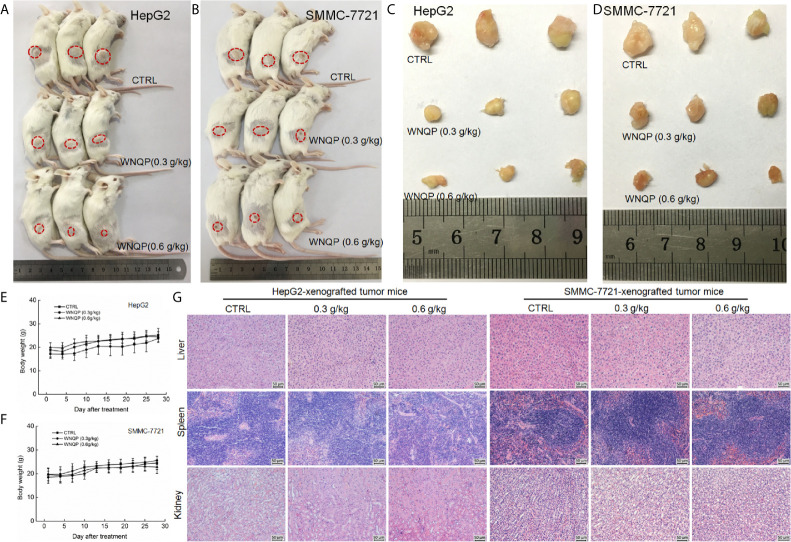
WNQP inhibited HepG2- and SMMC-7721-xenograft tumor growth in BALB/c mice. After three consecutive CTX injections, BALB/c mice inoculated with HepG2 and SMMC-7721 cells were treated with WNQP (0.3 and 0.6 g/kg dissolved in 0.9% saline solution) or vehicle solvent (0.9% saline solution) for 28 days. **(A, B)** Tumor-bearing mice and **(C, D)** tumor tissues from control (CTRL) and WNQP-treated groups (*n* = 3). **(E, F)** Mean (± SD) bodyweights of mice in the WNQP-treated and CTRL groups (*n* = 6). **(G)** H&E staining of liver, spleen, and kidney tissues from BALB/c mice (*n* = 3) (20× magnification, scale bar: 50 μm).

The BALB/c mice bearing tumor xenografts were used to investigate whether the effects of WNQP against liver cancer occur *via* the modulation of immune functions. A 28-day treatment regimen of WNQP administered at either 0.3 or 0.6 g/kg strongly inhibited the growth of HepG2-xenografted (**Figures 3A, C**) and SMMC-7721-xenografted tumors (**Figures 3B, D**), without any effects on bodyweights (**Figures 3E, F**) and organ structures of the liver, spleen, and kidney (**Figure 3G**).

**Figure 4:**
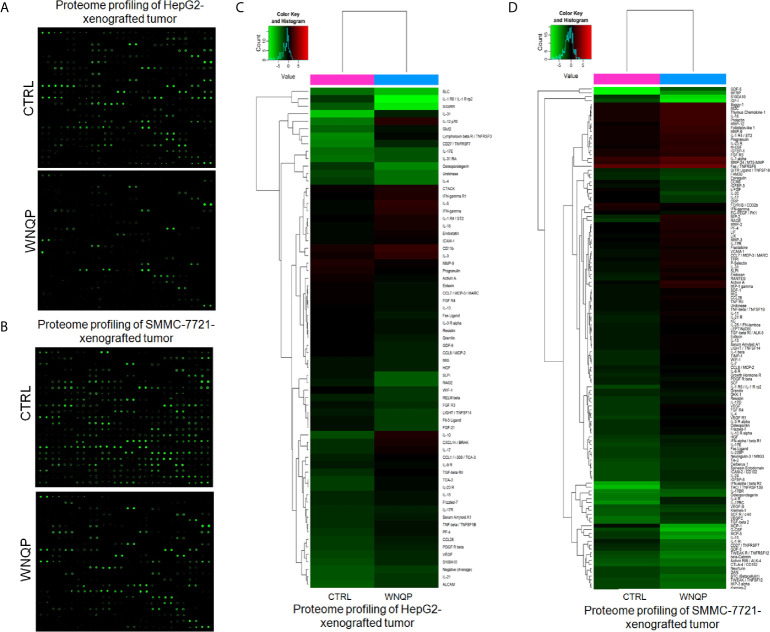
**(A, B)** The effects of WNQP on 308 types of cytokines in mice tumors, detected using the L-Series Mouse Antibody Array 308 Glass Slide Kit (*n* = 3). **(C, D)** Proteomic profiling of HepG2- and SMMC-7721-xenografted tumors. When compared with CTRL mice, WNQP-treated mice showed regulated levels of 66 types of cytokines in HepG2-xenografted tumors and of 57 types of cytokines in SMMC-7721-xenografted tumors.

A RayBio L-Series Mouse Antibody Array 308 Glass Slide Kit was used to detect 308 proteins in the tumor tissues. As compared to control mice, WNQP administered at 0.6 g/kg markedly affected the levels of 66 types of cytokines in the HepG2-xenografted tumors (**Figures 4A, C** and **Table S2**), and 57 types of cytokines in the SMMC-7721-xenografted tumors (**Figures 4B, D** and **Table S3**). Most of the cytokines were associated with immune functions.

Based on these results, seven types of immunological correlation factors were analyzed from the serum of BALB/c mice using ELISA. In BALB/c mice bearing HepG2- and SMMC-7721-xenografted tumors, WNQP enhanced the serum levels of TNF-α (*P <*0.05), TNF-β (*P <*0.05), IFN-γ (*P <*0.05), IL-2 (*P <*0.05), IL-10 (*P <*0.05), and CCL-28 (*P <*0.05), and reduced the serum levels of IL-31 (*P <*0.05) (**Table 2**).

**Table 2:** The effects of WNQP on the immune factors of serum in tumor-xenografted BALB/c mice.

Factors (pg/ml)	HepG2-xenografted tumor BALB/c mice			SMMC-7721-xenografted tumor BALB/c mice		
	CTRL	WNQP (0.3 g/kg)	WNQP (0.6 g/kg)	CTRL	WNQP (0.3 g/kg)	WNQP (0.6 g/kg)
TNF-α	784.7 ± 35.9	820.0 ± 27.8	858.1 ± 48.0*	773.8 ± 69.6	831.6 ± 18.0	850.8 ± 32.5*
TNF-β	297.3 ± 11.6	327.1 ± 29.1*	310.9 ± 12.5	251.2 ± 3.7	311.8 ± 16.3**	307.7 ± 38.9*
IFN-γ	249.2 ± 11.8	281.4 ± 18.8*	249.4 ± 16.8	257.5 ± 12.7	254.6 ± 21.5	260.6 ± 27.8
IL-2	421.7 ± 25.9	423.5 ± 43.1	413.2 ± 10.6	387.6 ± 12.1	430.9 ± 18.1*	468.5 ± 32.9*
IL-10	489.7 ± 39.8	545.5 ± 46.7*	589.7 ± 35.8*	417.2 ± 3.1	480.1 ± 36.2*	526.9 ± 54.5**
IL-31	50.1 ± 5.2	49.6 ± 3.2	41.9 ± 3.2*	51.7 ± 4.4	50.5 ± 6.1	49.2 ± 6.3
CCL-28 (*10^3^)	48.5 ± 2.3	62.6 ± 10.7*	66.0 ± 6.8**	44.9 ± 9.3	49.4 ± 5.2	54.5 ± 5.9*

Results are represented as means ± S.D. (n = 6). *P <0.05 and **P <0.01 vs. related control group, respectively.

### Nrf2 Signaling Is Involved in WNQP-Mediated Inhibition of Tumor Growth

**Figure 5:**
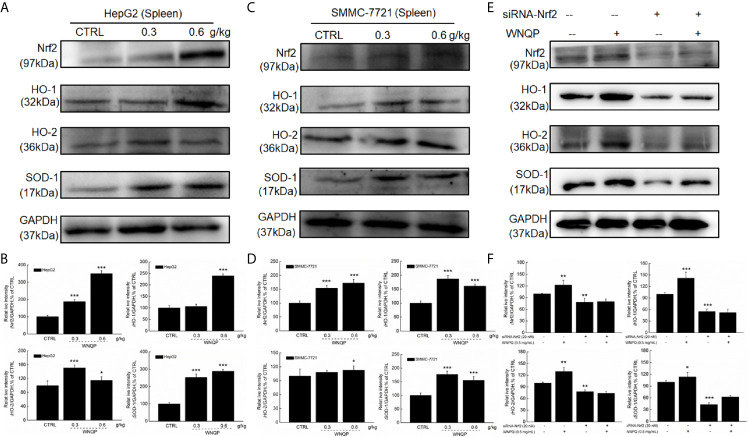
Nrf2 signaling is involved in the improvement of immune function during WNQP-mediated inhibition of tumor growth. WNQP strongly enhanced the expression levels of Nrf2, HO-1, HO-2 and SOD-1 in the spleen tissues of BALB/c mice bearing **(A, B)** HepG2- and **(C, D)** SMMC-7721-xenograft tumors. **(E, F)** In Nrf2-siRNA-transfected primary cultured spleen cells, WNQP-induced increase in the levels of Nrf2, HO-1, HO-2 and SOD-1 were abolished. Quantitative protein expression data were normalized to the corresponding GAPDH levels, and the average fold changes in band intensity are marked. Data are expressed as the mean ± SD (*n* = 3). *P < 0.05, **P < 0.01 and ***P < 0.001 *versus* the control group.

We used western blotting to analyze the changes in the expression of Nrf2 and its downstream factors in the spleen tissue of BALB/c mice. A 28-day treatment regimen of WNQP administration strongly enhanced the expression levels of Nrf2, HO-1, HO-2, and SOD-1 in the spleens of BALB/c mice carrying xenografts of both HepG2- (*P <*0.05) (**Figures 5A, B**) and SMMC-7721 tumors (*P <*0.05) (**Figures 5C, D**).

In primary cultured spleen cells transfected with Nrf2-siRNA, the WNQP failed to increase in the expression levels of Nrf2, HO-1, HO-2, and SOD-1 (**Figures 5E, F**) This suggested an important role of Nrf2 in the improvement of immune function during WNQP-mediated inhibition on tumor growth.

## Discussion

In this study, we first systematically confirmed the inhibitory effects of the Traditional Chinese Medicine formula, WNQP, on the growth of HepG2- and SMMC-7721-xenografted tumors in nude mice and BALB/c mice models. WNQP effectively inhibited tumor growth, but had no effects on the bodyweights and organ structures, confirming that it was safe to use in mice. Traditional Chinese Medicine plays an important role in the treatment of tumors. When combined with chemotherapy, radiation therapy, or post-operative treatment, Traditional Chinese Medicine can help to reduce toxic side effects (20).

Cancers are usually associated with uncontrolled cell proliferation and oxidative stress (21). As a physiological and/or pathological process, apoptosis has been recognized as a therapeutic target for various types of cancers; these therapeutic strategies commonly require the regulation of cytokines (22). As members of the Bcl-2 family, Bcl-2 and Bax are usually present as heterodimers and regulate the initiation of apoptosis by modulating mitochondrial activity that in turn regulates the release of cytochrome C and the caspase cascade (21). HSPs are a large family of molecular chaperones, which participate in the folding and maturation of a variety of client proteins and protect them from degradation, oxidative stress, hypoxia and heat stress (23). Abnormally high levels of HSP have been noted in tumor tissues. They participate in the caspase-mediated apoptotic pathway to protect mitochondrial integrity. High levels of HSP also enhance the resilience of tumors, allowing them to evade immune-mediated apoptosis (24). We observed that the levels of HSP27 and HSP60 were strongly suppressed after WNQP administration in nude mice. HSP27 can inhibit p53-mediated induction of p21, resulting in unlimited proliferation of tumor cells and eventually leading to tumorigenesis (25). HSP60, an immunodominant antigen associated with cellular immunity and immune responses to certain infectious diseases, prevents cellular apoptosis by inhibiting the activation of pro-apoptotic factors (26). HSP60 binds to Bax and Bcl-2, preventing Bax activation and maintaining normal folding of Bcl-2 to prevent apoptosis (27). HSP70 can enhance the immunosuppressant function of T regulatory cells (Tregs) and downregulate the secretion of cytokines IFN-γ and TNF-α (28, 29) Data obtained from nude mice bearing HepG2- and SMMC-7721-xenografted tumors confirmed that WNQP showed pro-apoptotic effects on liver cancers while inhibiting tumor growth. The apoptosis of tumor cells induced by WNQP has also been associated with immune regulation.

Carcinogenesis involves several important processes, including, but not limited to, migration, invasion, metastasis, and angiogenesis; all of these are dependent on the extracellular environment (30). The antibody chip array screen in immunosuppression BALB/c mice bearing a hepatoma showed that WNQP regulated the serum levels of interleukins and chemokines that can in turn regulate tumor progression. IL-10 has been shown to have anti-tumor effects, which are enhanced when combined with IL-2; these effects can potentially be harnessed for immunotherapy (31). IL-2 stimulates the growth of thymocytes and, as a result, induces T-cell differentiation and prompts the immune system to attack tumor cells (32). IL-2 can activate nature killer (NK) cells to secrete CCL28, thus enhancing the targeted killing of tumor cells. TNF can regulate immunity and enhance the anti-tumor cytolytic activity of NK cells and production of cytotoxicity-related proteins such as IFN-γ (33, 34). However, some cytokines can also promote tumor development (35). It has been reported that IL-31 can improve angiogenesis and promote tumor progression (36, 37). WNQP regulates these cytokines to form an immune regulatory network that inhibits the growth of tumor cells.

The activation of Nrf2 has been considered beneficial for the prevention of cancer, as Nrf2 is the main cellular defense mechanism against carcinogens, ROS, and other DNA-damaging factors (38). Activated Nrf2 can modulate cellular antioxidant regulators; it can upregulate the expression of HO-1, SOD-1, and other downstream genes in normal cells to maintain the intracellular redox balance and reduce tumorigenesis (39). Activated Nrf2 is involved in the regulation of immune factors (40, 41), which it achieves by inducing T lymphocytes to produce ILs, interferons, and TNF (42). In our previous experiments, we showed that *Antrodia cinnamomea* polysaccharides (APCS) enhance immune functions in mice by upregulating the expression of Nrf2 (43). Cumulatively, these results indicate that WNQP inhibits oxidative stress by regulating Nrf2 and its downstream proteins to counteract ROS generation and accumulation, increases SOD activity, regulates the levels of inflammatory factors and thus, further suppresses the growth of tumors in mice.

Some studies have reported that Nrf2 is frequently mutated in human cancer cells, leading to an increase in the expression of the corresponding protective genes and thereby giving these cells a growth advantage and anti-apoptotic ability (44). However, the activation of Nrf2 is a double-edged sword in the context of cancer (45). The expression of Nrf2 in cancer cells can promote tumor growth, while in the host cells it can limit tumor growth by maintaining a functional immune system (46, 47). Both Nrf2 inducers and inhibitors have been predicted to function as anti-cancer drugs, although their targets are different (48–51). Nrf2 inducers can protect normal cells from anticancer drugs during chemotherapy, implying that Nrf2 inducers in combination with anti-cancer drugs may help to overcome the limitations of traditional chemotherapy (44). This is consistent with our data showing that WNQP can be used for liver cancer treatment in combination with chemotherapeutics.

## Conclusion

WNQP inhibited the tumor growth of HepG2- and SMMC-7721-xenografted tumor models in nude mice and BALB/c mice. This effect is at least partially due to the regulation of oxidative stress-mediated immunomodulation. However, the study has some limitations. We failed to identify the key ingredient in WNQP that mediated the inhibition of liver cancer growth. This aspect needs further investigation.

## Data Availability Statement

The original contributions presented in the study are included in the article/**Supplementary Material**. Further inquiries can be directed to the corresponding authors.

## Ethics Statement

The experimental animal protocol was approved by the Institutional Animal Care and Use Committee of Jilin University.

## Author Contributions

YQ and YW designed the experiments, draft and revised the manuscript. XZ, XL, YuZ, and AY performed the experiments and analyzed the data. YoZ and ZT analyzed the data. All authors contributed to the article and approved the submitted version.

## Funding

This work was supported by the Special Projects of Cooperation between Jilin University and Jilin Province in China (SXGJSFKT2020-1), Medical Health Project in Jilin Province of P. R. China (Grant No.20191102027YY, 20200708037YY and 20200708068YY).

## Conflict of Interest

Author ZT was employed by Jilin Tianlitai Pharmaceutical Co. Ltd.

The remaining authors declare that the research was conducted in the absence of any commercial or financial relationships that could be construed as a potential conflict of interest.

## Abbreviations

ARE, antioxidant response element; Bax, bcl2-associated X; Bcl-xL, B-cell lymphoma-extra-large; bZIP, basic leucine zipper; CCL, chemokine (C–C motif) ligand; ELISA, enzyme-linked immunosorbent assay; FADD, Fas-associated protein with death domain; GAPDH, glyceraldehyde-3-phosphate dehydrogenase; HO-1, heme oxygenase-1; HRP, horseradish peroxidase; HSP, heat shock protein; H&E, hematoxylin and eosin; IFN, interferon; IL, interleukin; MMP, matrix metalloproteinase; Nrf2, nuclear factor-erythroid 2-related factor 2; PFA, paraformaldehyde; ROS, reactive oxygenated species; SOD, superoxide dismutase; SPF, specific pathogen-free; TNF, tumor necrosis factor; WNQP, Wan-Nian-Qing-Prescription.
