# Supplementary material for: Wan-Nian-Qing, a Herbal Composite Prescription, Suppresses the Progression of Liver Cancer in Mice by Regulating Immune Response
Source: Front Oncol. 2021 Jul 8;11:696282. doi: 10.3389/fonc.2021.696282 (PMC8297951; doi:10.3389/fonc.2021.696282)
Supplement: Supplementary file 1 [file Table_1.doc]

Table.1s. The data of the Proteome Profler^TM^ Array using the Human Apoptosis Array Kit

| Coordinate | Target | HepG2-xenograftedtumor SMMC-7721-xenografted tumor | | | | | |
| --- | --- | --- | --- | --- | --- | --- | --- |
|  |  | Pixel Density | | Fold | Pixel Density | | Fold |
|  |  | CTRL | Treated |  | CTRL | Treated |  |
| B1,B2 | Bad | 0.3628 | 0.3715 | 1.02 | 0.3836 | 0.3890 | 1.01 |
| B3,B4 | Bax | **0.2824** | **0.3450** | **1.22** | 0.3302 | 0.2792 | 0.85 |
| B5,B6 | Bcl-2 | 0.1697 | 0.1981 | 1.17 | **0.1918** | **0.1550** | **0.81** |
| B7,B8 | Bcl-x | 0.0863 | 0.0816 | 0.95 | **0.1015** | **0.0654** | **0.64** |
| B9,B10 | Pro-Caspase-3 | 0.8727 | 0.8348 | 0.96 | 0.8959 | 0.7934 | 0.89 |
| B11,B12 | Cleaved Caspase-3 | 0.3628 | 0.3218 | 0.89 | **0.5288** | **0.1916** | **0.36** |
| B13,B14 | Catalase | 0.8124 | 0.7807 | 0.96 | 0.7599 | 0.7800 | 1.03 |
| B15,B16 | cIAP-1 | 0.2960 | 0.2663 | 0.90 | **0.2690** | **0.2094** | **0.78** |
| B17,B18 | cIAP-2 | 0.1915 | 0.2156 | 1.13 | 0.1940 | 0.1764 | 0.91 |
| B19,B20 | Claspin | 0.2442 | 0.2526 | 1.03 | 0.2405 | 0.2012 | 0.84 |
| B21,B22 | Clusterin | 0.3610 | 0.2862 | 0.79 | **0.3116** | **0.1662** | **0.53** |
| B23,B24 | Cytochrome c | 0.2261 | 0.2217 | 0.98 | 0.2499 | 0.1812 | 0.73 |
| C1,C2 | TRAIL R1/DR4 | 0.1980 | 0.1881 | 0.95 | 0.2135 | 0.1856 | 0.87 |
| C3,C4 | TRAIL R2/DR5 | 0.2955 | 0.2604 | 0.88 | **0.3065** | **0.2421** | **0.79** |
| C5,C6 | FADD | 0.4114 | 0.3463 | 0.84 | **0.5056** | **0.2927** | **0.58** |
| C7,C8 | Fas/TNFRSF6/CD95 | 0.6002 | 0.3779 | 0.63 | **0.5426** | **0.3155** | **0.58** |
| C9,C10 | HIF-1α | 0.3885 | 0.2824 | 0.73 | **0.3708** | **0.2433** | **0.66** |
| C11,C12 | HO-1/HMOX1/HSP32 | 0.3608 | 0.3048 | 0.84 | **0.3918** | **0.2648** | **0.68** |
| C13,C14 | HO-2/HMO2 | 0.5974 | 0.5257 | 0.88 | 0.5314 | 0.4470 | 0.84 |
| C15，C16 | HSP27 | 0.6688 | 0.6680 | 1.00 | 0.6588 | 0.6224 | 0.94 |
| C17,C18 | HSP60 | 0.5396 | 0.5699 | 1.06 | **0.5862** | **0.3049** | **0.52** |
| C19,C20 | HSP70 | 0.6705 | 0.6874 | 1.03 | 0.6491 | 0.6345 | 0.98 |
| C21,C22 | HTRA2/Omi | 0.2501 | 0.2130 | 0.85 | **0.1887** | **0.1489** | **0.79** |
| C23,C24 | Livin | 0.1522 | 0.1407 | 0.92 | **0.1588** | **0.1216** | **0.77** |
| D1,D2 | PON2 | 0.2597 | 0.2543 | 0.98 | 0.2633 | 0.2330 | 0.89 |
| D3,D4 | p21/CIP1/CDKN1A | 0.1985 | 0.2359 | 1.19 | 0.2108 | 0.1901 | 0.90 |
| D5,D6 | p27/Kip1 | 0.1903 | 0.1750 | 0.92 | 0.1915 | 0.1784 | 0.93 |
| D7,D8 | Phospho-p53 (S15) | 0.2240 | 0.2186 | 0.98 | 0.2194 | 0.1831 | 0.83 |
| D9,D10 | Phospho-p53 (S46) | 0.1954 | 0.2017 | 1.03 | 0.1892 | 0.1582 | 0.84 |
| D11,D12 | Phospho-p53 (S392) | 0.1272 | 0.1334 | 1.05 | 0.1247 | 0.1026 | 0.82 |
| D13,D14 | Phospho-Rad17(S635) | 0.2323 | 0.2099 | 0.90 | 0.2063 | 0.2082 | 1.01 |
| D15,D16 | SMAC/Diablo | 0.6257 | 0.5476 | 0.88 | **0.5848** | **0.4525** | **0.77** |
| D17,D18 | Survivin | 0.7261 | 0.5885 | 0.81 | **0.6917** | **0.5013** | **0.72** |
| D19,D20 | TNF RI/TNFRSF1A | 0.2021 | 0.2065 | 1.02 | 0.1788 | 0.1588 | 0.89 |
| D21,D22 | XIAP | 0.2389 | 0.2218 | 0.93 | **0.1887** | **0.1450** | **0.77** |

Table.2s. The data of the RayBio L-Series Mouse Antibody Array 308 Glass Slide Kit (HepG2-xenograftedtumor)

| Coordinate | | Target | HepG2-xenograftedtumor | | | |
| --- | --- | --- | --- | --- | --- | --- |
| Row | Column |  | CTRL | | Treated | Fold |
| 1 | 11~12 | Activin A | | 0.7670 | 1.4370 | 1.8735 |
| 2 | 15~16 | BLC | | 0.0075 | 0.0542 | 7.2703 |
| 2 | 27~28 | CCL7 / MCP-3 / MARC | | 0.7347 | 1.1312 | 1.5398 |
| 3 | 1~2 | CCL8 / MCP-2 | | 0.4388 | 0.9218 | 2.1008 |
| 6 | 11~12 | Eotaxin | | 0.7285 | 1.2156 | 1.6687 |
| 7 | 1~2 | Fas Ligand | | 0.5768 | 0.9354 | 1.6217 |
| 7 | 5~6 | FGF R3 | | 0.2946 | 0.6191 | 2.1013 |
| 7 | 7~8 | FGF R4 | | 0.6762 | 1.1538 | 1.7062 |
| 7 | 11~12 | FGF-21 | | 0.2238 | 0.6341 | 2.8341 |
| 7 | 13~14 | Fit-3 Ligand | | 0.2325 | 0.6266 | 2.6956 |
| 8 | 11~12 | GDF-9 | | 0.4500 | 1.0695 | 2.3766 |
| 9 | 5~6 | Gremlin | | 0.6116 | 1.0213 | 1.6698 |
| 9 | 11~12 | HGF | | 0.4376 | 0.7441 | 1.7005 |
| 10 | 27~28 | IL-1 R6 / IL-1 R rp2 | | 0.0012 | 0.2440 | 196.2979 |
| 11 | 15~16 | IL-3 R alpha | | 0.6414 | 0.9670 | 1.5076 |
| 11 | 19~20 | IL-4 | | 0.0522 | 0.2139 | 4.0968 |
| 13 | 3~4 | IL-13 | | 0.6762 | 1.0951 | 1.6193 |
| 15 | 23~24 | LIGHT / TNFSF14 | | 0.2250 | 0.6040 | 2.6845 |
| 16 | 23~24 | MIG | | 0.4624 | 0.7215 | 1.5602 |
| 17 | 11~12 | MMP-9 | | 1.1238 | 2.0365 | 1.8122 |
| 18 | 3~4 | Osteoporotegerin | | 0.0323 | 0.2350 | 7.2703 |
| 18 | 19~20 | Progranulin | | 1.0355 | 1.7684 | 1.7077 |
| 18 | 25~26 | RAGE | | 0.0932 | 0.5769 | 6.1878 |
| 19 | 1~2 | RELM beta | | 0.2362 | 0.4594 | 1.9451 |
| 19 | 3~4 | Resistin | | 0.6178 | 1.0695 | 1.7310 |
| 19 | 17~18 | SIGIRR | | 0.0025 | 0.1024 | 41.1983 |
| 19 | 19~20 | SLPI | | 0.0920 | 0.6643 | 7.2212 |
| 22 | 21~22 | Urokinase | | 0.0597 | 0.1913 | 3.2060 |
| 23 | 13~14 | WIF-1 | | 0.2772 | 0.9294 | 3.3526 |
| 1 | 21~22 | ALCAM | | 0.2424 | 0.1054 | 0.4350 |
| 2 | 21~22 | CCL1 / I-309 / TCA-3 | | 1.0318 | 0.4654 | 0.4511 |
| 2 | 23~24 | CCL28 | | 0.5395 | 0.1943 | 0.3602 |
| 3 | 15~16 | CD11b | | 4.1793 | 1.9582 | 0.4685 |
| 3 | 21~22 | CD27 / TNFRSF7 | | 0.3767 | 0.0362 | 0.0960 |
| 4 | 23~24 | CTACK | | 2.3507 | 1.2894 | 0.5485 |
| 4 | 27~28 | CXCL14 / BRAK | | 1.7329 | 0.3118 | 0.1799 |
| 6 | 9~10 | Endostatin | | 1.4669 | 0.8842 | 0.6028 |
| 7 | 25~26 | Frizzled-7 | | 0.7061 | 0.3464 | 0.4907 |
| 8 | 23~24 | Glut2 | | 0.6377 | 0.0783 | 0.1228 |
| 9 | 15~16 | ICAM-1 | | 1.2667 | 0.8194 | 0.6469 |
| Continued | | | | | | |
| Coordinate | | Target | HepG2-xenograftedtumor | | | |
| Row | Column |  | CTRL | | Treated | Fold |
| 10 | 1~2 | IFN-gamma | | 2.6279 | 0.7486 | 0.2849 |
| 10 | 3~4 | IFN-gamma R1 | | 2.1630 | 1.0574 | 0.4889 |
| 10 | 25~26 | IL-1 R4 / ST2 | | 1.6359 | 0.9595 | 0.5865 |
| 11 | 13~14 | IL-3 | | 3.4446 | 1.4249 | 0.4137 |
| 11 | 23~24 | IL-5 | | 2.9785 | 0.8028 | 0.2696 |
| 12 | 17~18 | IL-9 R | | 0.7807 | 0.5091 | 0.6522 |
| 12 | 19~20 | IL-10 | | 2.2599 | 0.1431 | 0.0633 |
| 12 | 27~28 | IL-12 p70 | | 2.8541 | 0.0482 | 0.0169 |
| 13 | 7~8 | IL-15 | | 0.6588 | 0.4037 | 0.6127 |
| 13 | 11~12 | IL-16 | | 1.6981 | 0.8812 | 0.5189 |
| 13 | 13~14 | IL-17 | | 1.5663 | 0.3073 | 0.1962 |
| 13 | 21~22 | IL-17E | | 0.1094 | 0.0723 | 0.6609 |
| 13 | 25~26 | IL-17R | | 0.4923 | 0.3223 | 0.6548 |
| 14 | 9~10 | IL-21 | | 0.2635 | 0.1115 | 0.4230 |
| 14 | 19~20 | IL-23 R | | 0.8801 | 0.2139 | 0.2430 |
| 14 | 27~28 | IL-31 | | 0.4997 | 0.0060 | 0.0121 |
| 15 | 1~2 | IL-31 RA | | 0.1094 | 0.0572 | 0.5232 |
| 16 | 7~8 | Lymphotoxin beta R / TNFRSF3 | | 0.7570 | 0.0301 | 0.0398 |
| 18 | 11~12 | PDGF R beta | | 0.4500 | 0.2094 | 0.4653 |
| 18 | 15~16 | PF-4 | | 0.6290 | 0.2802 | 0.4454 |
| 19 | 5~6 | S100A10 | | 0.3468 | 0.1431 | 0.4126 |
| 19 | 13~14 | Serum Amyloid A1 | | 0.5743 | 0.3133 | 0.5455 |
| 20 | 3~4 | TCA-3 | | 0.9000 | 0.2094 | 0.2326 |
| 20 | 19~20 | TGF-beta RII | | 0.9311 | 0.2952 | 0.3171 |
| 21 | 23~24 | TNF-beta / TNFSF1B | | 0.6464 | 0.2982 | 0.4614 |
| 22 | 27~28 | VEGF | | 0.3667 | 0.1702 | 0.4641 |

Table.3s. The data of the RayBio L-Series Mouse Antibody Array 308 Glass Slide Kit (SMMC-7721-xenograftedtumor)

| Coordinate | | Target | SMMC-7721-xenograftedtumor | | | | | |  | |
| --- | --- | --- | --- | --- | --- | --- | --- | --- | --- | --- |
| Row | Column |  | CTRL | | Treated | | Fold | |  | |
| 1 | 11~12 | Activin A | | 2.9126 | | 0.8266 | | 0.2838 | | |
| 2 | 27~28 | CCL7 / MCP-3 / MARC | | 1.7358 | | 0.7548 | | 0.4349 | | |
| 6 | 5~6 | Endocan | | 1.6525 | | 0.6172 | | 0.3735 | | |
| 6 | 11~12 | Eotaxin | | 1.1734 | | 0.5717 | | 0.4872 | | |
| 7 | 7~8 | FGF R4 | | 0.9998 | | 0.3149 | | 0.3150 | | |
| 7 | 17~18 | Follistatin-like 1 | | 3.4854 | | 1.3256 | | 0.3803 | | |
| 7 | 25~26 | Frizzled-7 | | 0.8991 | | 0.3702 | | 0.4117 | | |
| 8 | 7~8 | GDF-5 | | 0.1319 | | 0.0039 | | 0.0294 | | |
| 9 | 17~18 | ICAM-2 / CD102 | | 0.4062 | | 0.1783 | | 0.4390 | | |
| 9 | 23~24 | IFN-alpha / beta R1 | | 0.5693 | | 0.2597 | | 0.4561 | | |
| 9 | 25~26 | IFN-alpha / beta R2 | | 0.2847 | | 0.0242 | | 0.0851 | | |
| 10 | 13~14 | IGFBP-6 | | 0.4149 | | 0.1986 | | 0.4788 | | |
| 10 | 23~24 | IL-1 beta | | 1.2272 | | 0.4215 | | 0.3435 | | |
| 10 | 25~26 | IL-1 R4 / ST2 | | 2.9265 | | 1.4215 | | 0.4857 | | |
| 10 | 27~28 | IL-1 R6 / IL-1 R rp2 | | 0.9113 | | 0.2209 | | 0.2424 | | |
| 11 | 15~16 | IL-3 R alpha | | 1.0276 | | 0.3585 | | 0.3489 | | |
| 11 | 19~20 | IL-4 | | 0.9200 | | 0.2897 | | 0.3149 | | |
| 11 | 21~22 | IL-4 R | | 0.4739 | | 0.0698 | | 0.1472 | | |
| 12 | 11~12 | IL-7 | | 1.0658 | | 0.4370 | | 0.4100 | | |
| 12 | 21~22 | IL-10 R alpha | | 0.8523 | | 0.3459 | | 0.4059 | | |
| 12 | 23~24 | IL-11 | | 1.2897 | | 0.6192 | | 0.4801 | | |
| 13 | 11~12 | IL-16 | | 3.7111 | | 1.6134 | | 0.4347 | | |
| 13 | 15~16 | IL-17BR | | 0.1371 | | 0.0610 | | 0.4452 | | |
| 13 | 19~20 | IL-17D | | 0.7394 | | 0.2888 | | 0.3905 | | |
| 13 | 21~22 | IL-17E | | 0.5294 | | 0.2238 | | 0.4228 | | |
| 13 | 27~28 | IL-17RC | | 0.5381 | | 0.0417 | | 0.0774 | | |
| 14 | 11~12 | IL-21 R | | 1.2810 | | 0.5252 | | 0.4100 | | |
| 14 | 21~22 | IL-24 | | 0.4149 | | 0.1986 | | 0.4788 | | |
| 14 | 27~28 | IL-31 | | 1.5848 | | 0.6919 | | 0.4366 | | |
| 15 | 9~10 | KC | | 1.2150 | | 0.5446 | | 0.4482 | | |
| 15 | 25~26 | LIX | | 1.8555 | | 0.8953 | | 0.4825 | | |
| 16 | 15~16 | M-CSF | | 2.6123 | | 1.1483 | | 0.4396 | | |
| 16 | 17~18 | MDC | | 3.9350 | | 1.6531 | | 0.4201 | | |
| 16 | 21~22 | MFRP | | 0.0382 | | 0.0029 | | 0.0761 | | |
| 16 | 27~28 | MIP-1 gamma | | 2.4422 | | 0.6996 | | 0.2865 | | |
| 17 | 1~2 | MIP-2 | | 2.2739 | | 0.4283 | | 0.1884 | | |
| 17 | 11~12 | MMP-9 | | 3.3813 | | 1.4312 | | 0.4233 | | |
| 17 | 13~14 | MMP-12 | | 3.6573 | | 1.2674 | | 0.3466 | | |
| 18 | 1~2 | Osteopontin | | 0.9668 | | 0.3721 | | 0.3849 | | |
| 18 | 19~20 | Progranulin | | 2.7998 | | 1.3643 | | 0.4873 | | |
| Continued | | | | | | | | | |  |
| Coordinate | | Target | SMMC-7721-xenograftedtumor | | | | | |  | |
| Row | Column |  | CTRL | | Treated | | Fold | |  | |
| 18 | 21~22 | Prolactin | | 4.1728 | | 1.7665 | | 0.4233 | | |
| 18 | 23~24 | P-Selectin | | 1.8972 | | 0.6938 | | 0.3657 | | |
| 18 | 25~26 | RAGE | | 1.8295 | | 0.2810 | | 0.1536 | | |
| 18 | 27~28 | RANTES | | 1.5153 | | 0.5523 | | 0.3645 | | |
| 19 | 3~4 | Resistin | | 0.6717 | | 0.3324 | | 0.4948 | | |
| 19 | 9~10 | SCF R / c-kit | | 0.3715 | | 0.1211 | | 0.3261 | | |
| 19 | 21~22 | Soggy-1 | | 4.0739 | | 1.6211 | | 0.3979 | | |
| 19 | 27~28 | TACI / TNFRSF13B | | 0.2743 | | 0.0136 | | 0.0495 | | |
| 20 | 9~10 | TFPI | | 1.6264 | | 0.8101 | | 0.4981 | | |
| 20 | 13~14 | TGF-beta 2 | | 0.2673 | | 0.0940 | | 0.3516 | | |
| 20 | 23~24 | Thymus Chemokine-1 | | 3.7736 | | 1.7345 | | 0.4596 | | |
| 20 | 27~28 | TIMP-1 | | 1.0311 | | 0.4128 | | 0.4004 | | |
| 22 | 23~24 | VCAM-1 | | 1.7983 | | 0.7723 | | 0.4295 | | |
| 22 | 27~28 | VEGF | | 1.2359 | | 0.3159 | | 0.2556 | | |
| 23 | 1~2 | VEGF R1 | | 0.8557 | | 0.2694 | | 0.3148 | | |
| 23 | 9~10 | VEGFC | | 0.3090 | | 0.0824 | | 0.2666 | | |
| 23 | 13~14 | WIF-1 | | 1.0345 | | 0.4525 | | 0.4374 | | |
